# Outcomes of Nephron Sparing in a Specialist Cancer Hospital of a Developing Country

**DOI:** 10.7759/cureus.4150

**Published:** 2019-02-27

**Authors:** Muhammad Arshad Irshad Khalil, Nouman Khan, Azfar Ali, Muhammad Abu Bakar, Siddique Adnan, Shaukat Fiaz, Aleena Akbar Khan, Khurram Mir

**Affiliations:** 1 Surgical Oncology, Shaukat Khanum Memorial Cancer Hospital and Research Center, Lahore, PAK; 2 Biostatistics and Epidemiology, Shaukat Khanum Memorial Cancer Hospital and Research Center, Lahore, PAK

**Keywords:** renal cell carcinoma, clavien-dindo surgical complications, nephron-sparing surgery, radical nephrectomy, partial nephrectomy, renal score

## Abstract

Introduction

Nephron-sparing surgery in the form of partial nephrectomy (PN) is currently considered the standard treatment for relatively small localized renal cell tumors.

Objectives

This study aimed to determine outcomes of PN regarding complications, recurrence, and survival rates at Shaukat Khanum Memorial Cancer Hospital and Research Centre, Lahore, Pakistan.

Methods

We assessed the data of patients older than 18 years undergoing PN from January 2010 to June 2017 who met our inclusion criteria. Data were analyzed using IBM SPSS Statistics for Windows, Version 20.0 (IBM Corp., Armonk, NY).

Results

A total of 35 patients were studied, with a male to female ratio of 2.5:1 with median age of 50 years. The median hospital stay was four days (range: 3-7), and the median RENAL (radius, exophytic/endophytic properties, nearness of tumor to the collecting system or sinus in millimeters, anterior/posterior location relative to polar lines) Score was five (range: 4-10). The most common pathological tumor stage was T1 (94%), and the median size was 3.5 cm. On histopathology, clear-cell carcinoma was the most common tumor (incidence, 71%). The median Fuhrman’s grade was two. On final histopathology, four patients had positive margins. Among them, two patients showed a progressive deterioration in renal functions and were found to have residual disease six months later. Only one patient developed metastasis in the lung. Wound infection was observed in one patient while another had wound dehiscence. Urine leakage was noted in two patients. The median follow-up duration was 18 months (range: 3-84). Mean cancer-free survival was 78.6 months, and overall survival was 79.2 months. The projected three-year and five-year disease-free and overall survival was 96% and 94%, respectively.

Conclusion

PN is a viable option with excellent outcomes regarding the complication profile, recurrence-free, and overall survival in patients with relatively small localized renal tumors.

## Introduction

Renal cell carcinoma (RCC) comprises 2% to 3% of all adult malignancies [1]. Its incidence is increasing for yet unknown reasons by almost 2.5% per year [2]. It is the ninth and 14th most common type of cancer in men and women, respectively, and the 16th leading cause of death from cancer. An increase in the incidence has also been observed in Asian countries. A total of 121,099 kidney cancer cases were recorded in Asian countries in 2012. China, Japan, and India are the three Asian countries with the highest incidence [3]. However, no authentic statistics on the incidence of RCC are available from Pakistan, which would require a large-scale enrollment of renal cancer care centers.

Given the availability and widespread use of modern imaging, very few patients present with the classical triad of loin pain, mass, and hematuria. Most cases of RCC are picked up incidentally [4] by common imaging such as ultrasound, computed tomography (CT) or magnetic resonance imaging for unrelated reasons. Among Stage 1 tumors, there is an increase in the incidence of small tumors (<2 cm) and 2-cm to 4-cm tumors [5]. This increase in incidence suggests the need for newer treatment approaches to RCC in addition to radical nephrectomy (RN), as first described by Robson et al. [6]. The most widely popularized surgical treatment option is nephron-sparing surgery (NSS) by partial nephrectomy (PN). This procedure has been investigated extensively and is the standard of care for smaller renal tumors [7].

PN was reserved for patients with solitary functioning kidney, bilateral renal tumors, hereditary renal tumors (e.g., Von Hippel-Lindau disease), patients with renal impairment, and those with comorbidities predisposing them to renal compromise in future. PN is now performed routinely and electively for patients with small tumors and healthy contralateral kidneys [8].

As a result, the number of patients with small renal tumors undergoing PN is steadily increasing. Experience with this surgical technique is accumulating globally, leading to satisfactory results in the form of better survival [9] and preservation of renal functions [10]. A similar trend has also been observed in our country. Many incidentally diagnosed small renal tumors are treated via PN, although factors like the acquisition of adequate skills and facilities like frozen section (FS) histology will certainly set the pace for this process becoming a common treatment approach. The history of PN in our institution differs little from the rest of the country. The first procedure was performed in June 2010. Selection of patients suitable for partial rather than radical nephrectomy has been an issue of debate, and the simple logic of small renal tumors does not seem to be sufficient for both the urologists and patients to be satisfied with this decision. As a result, scientific, logical, and evidenced-based decision criteria are needed. The RENAL (radius, exophytic/endophytic properties, nearness of tumor to the collecting system or sinus in millimeters, anterior/posterior location relative to polar lines) nephrometric scoring system is one recently used tool and is based on cross-sectional radiographic characteristics of the tumor. This system was first introduced by Alexander Kutikov to objectively relate the tumor's anatomical features to its complexity and ease of resection [11]. Its utility is highlighted in several studies [12]. It can be utilized to decide between RN and PN procedures objectively. This scoring system utilizes five variables: radius (in cm), endophytic or exophytic component, nearness to the pelvicalyceal system, anterior or posterior location, and location relative to the polar lines to assign a score to the tumor. A tumor can have a minimum RENAL score of four and a maximum score of 12. The higher the score, the more complex the tumor and therefore, the less likely the tumor could undergo PN. RENAL scoring is classified into low (four to six), moderate (seven to nine) and high (10 to 12) complexities. Additionally, more complex renal lesions are more prone to develop postoperative complications [13]. In this article, we present our initial experience of PN and the overall and recurrence-free survival of patients undergoing this procedure.

## Materials and methods

We retrieved the medical records of all patients undergoing treatment for renal cancer from the hospital information system. To be included in the study, the records had to be for patients aged 18 or older with solitary tumors undergoing PN at the department of surgical oncology at the Shaukat Khanum Memorial Cancer Hospital and Research Center in Lahore, Pakistan from January 2010 to June 2017. Patients with a familial predisposition to renal cancer development were excluded to counter the bias for local recurrence versus de novo tumor redevelopment. Patients with a history of radical or PN on the contralateral side in the previous five years were also excluded to assess the results of PN for a particular tumor and to offset the effect of previous cancer history. We analyzed patient demographics, medical comorbidities, presenting concerns, and preoperative and postoperative tumor characteristics.

Statistical analysis was carried out using IBM SPSS Statistics for Windows, Version 20.0 (IBM Corp., Armonk, NY). Continuous variables were stated as mean ± standard deviation, and categorical variables were computed as frequencies and percentages. The Kaplan-Meier method was used to estimate survival. Formal approval for the study was obtained from the Institutional Review Board before commencing data collection.

## Results

A total of 43 patients underwent PN during the study period, and of those, eight were excluded based on the exclusion criteria. The mean age and standard deviation of the patients was 48 ± 13 years. The study sample had 25 male and 10 female patients. We had 18 patients with right-sided tumors, and 17 had left-sided tumors. Regarding comorbidities, 25.7% of the patients had diabetes, and 28.6% had hypertension treated with medication. Two patients had deranged renal functions preoperatively. Seven patients (20%) were cigarette smokers. Patients’ demographic characteristics are presented in Table 1. The main presenting concern was pain in 14 patients (40.9%). We found no hematuria, fever or weight loss.

**Table 1:** Patient demographic characteristics and comorbidities

Demographic information	
Age (years; mean ± SD)	48.11 ± 13.23
Patient characteristics	N (%)
Ethnicity	
Khyber Pakhtunkhwa	10 (28.5%)
Punjab	24 (68.5%)
Sindh	1 (2.8%)
Gender	
Male	25 (71.4%)
Female	10 (28.5%)
Tumor location	
Right side	18 (51.4%)
Left side	17 (48.5%)
Diabetes	
No	26 (74.28%)
Yes	9 (25.71%)
Hypertension	
No	25 (71.4%)
Yes	10 (28.5%)
Smoker	
No	28 (80%)
Yes	7 (20%)

Furthermore, 20 patients (57.1%) had a tumor incidentally diagnosed during an investigation for non-renal concerns (Table 2). PN was performed through a loin incision in 20 patients, and anterior subcostal incision was made in 15 patients. The tumor was located at the upper pole in nine patients, the middle pole in 13 patients, and the lower pole in 13 patients. The median blood loss was 100 mL, the median hospital stay was four days. The median follow-up duration was 18 months (range: 3-84).

**Table 2:** Presenting concerns

Concern	N (%)
Pain	14 (40%)
Lower Urinary Tract Symptoms	1 (2.8%)
Incidental	20 (57%)

**Figure 1:**
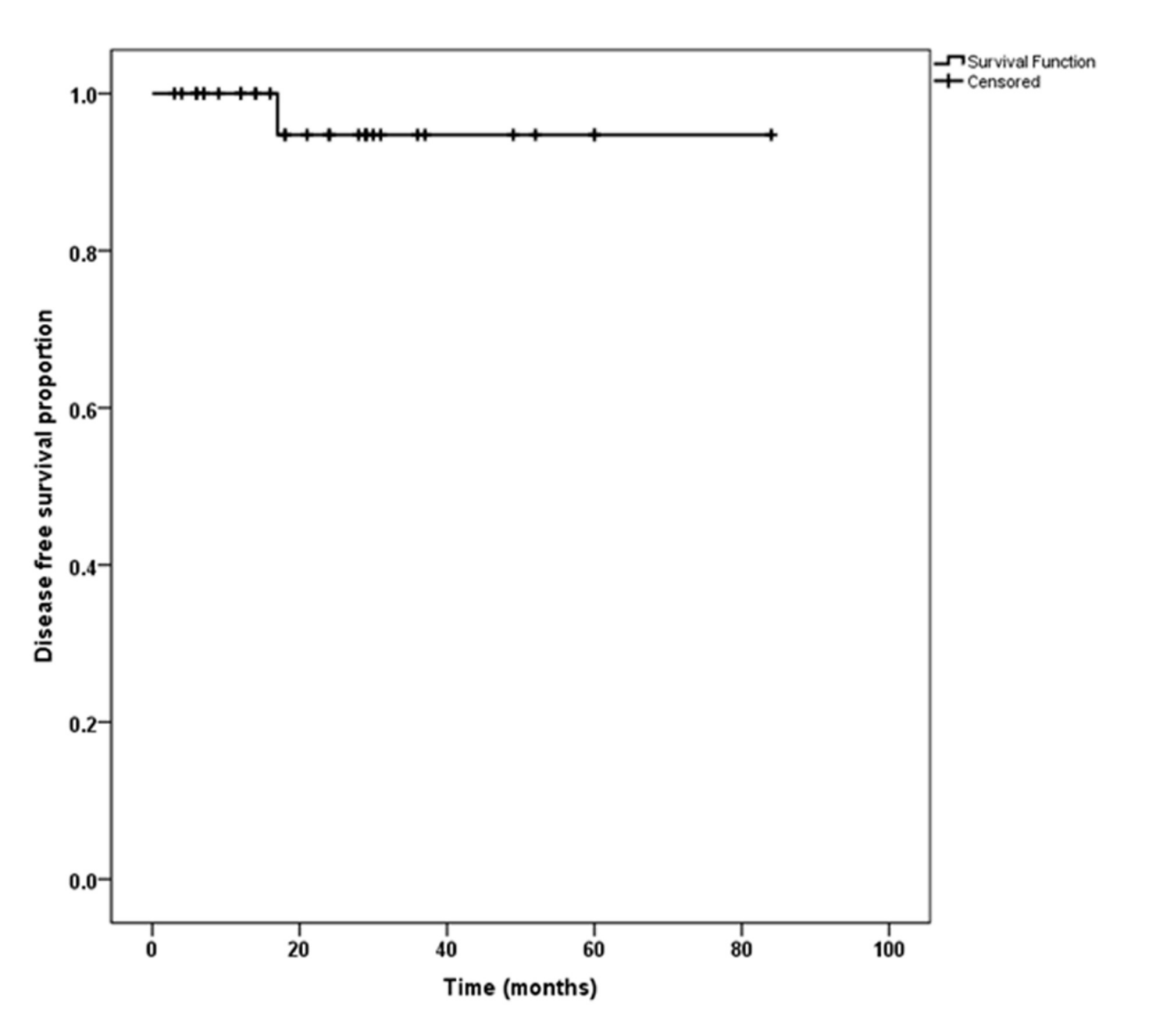
Post partial nephrectomy recurrence-free survival

**Figure 2:**
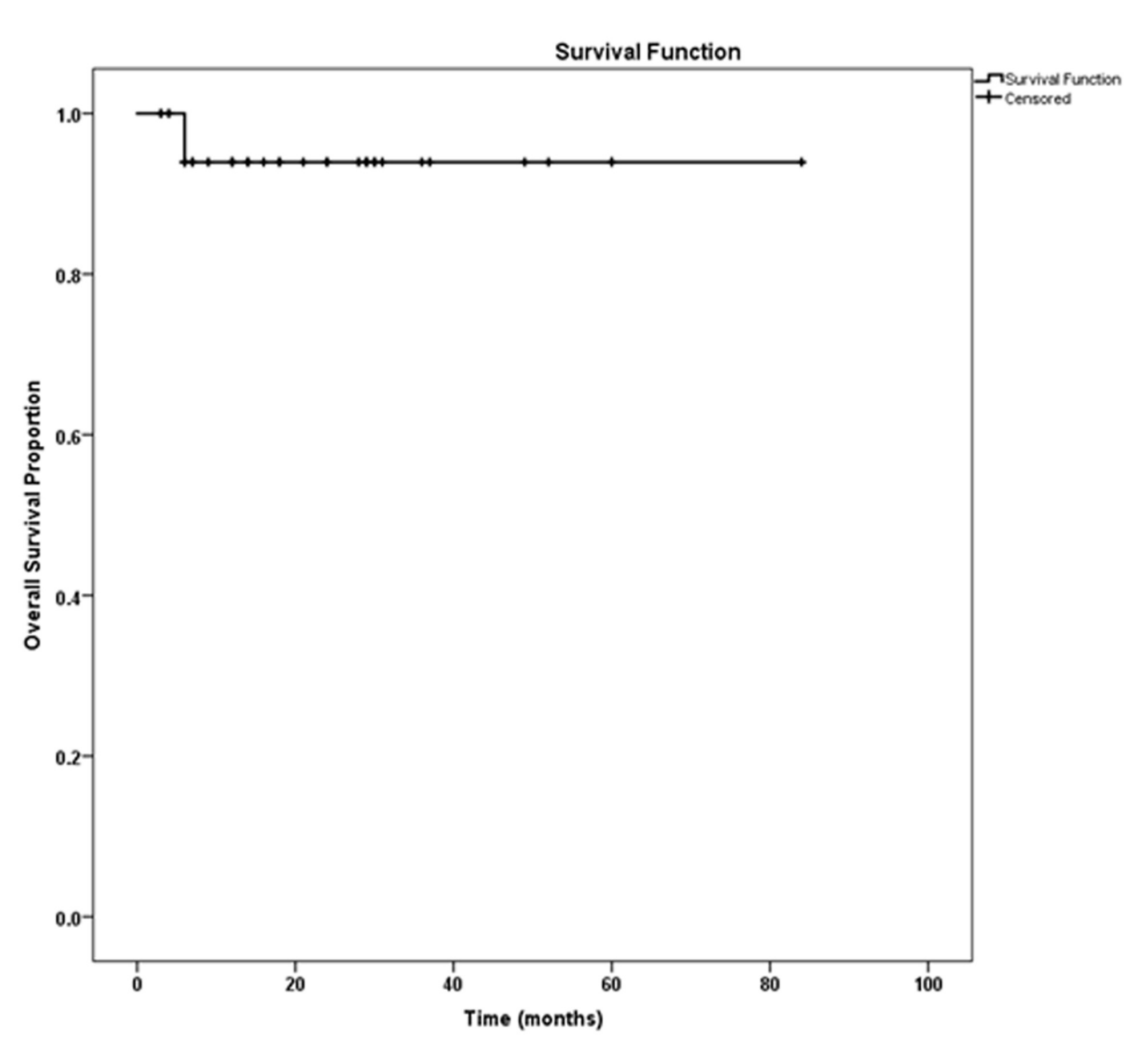
Post partial nephrectomy overall survival

On analysis, the mean cancer-free survival was 78.6 ± 5.12 months (Figure 1) while the overall survival was 79.2 ± 3.24 months (Figure 2). The projected five-year disease-free survival and overall survival was calculated to be 96% and 94%, respectively. The most common pathological tumor stage was Stage T1a present in 25 patients, followed by T1b in eight patients, and T3 in two patients. The median tumor size was 3.5 cm.

On histopathologic examination, RCC was the most common finding (33 patients). On further analysis, 25 patients had clear cell variant of RCC, six patients had papillary, and two had a multiloculated variant of RCC. Moreover, one patient had an angiomyolipoma, and another was diagnosed with a pseudotumor. The median RENAL score was five (range: 4-10) and the median Fuhrman’s grade was two (range: 1-5). None of the tumors had a lymphovascular invasion.

We performed a histological examination of FS from 32 patients and found two positive specimens for which additional resection was performed and negative margins were achieved. On final histopathology, four patients had a positive margin. Among them, FS histology was not performed in three patients. Positive margin despite a negative FS was observed in one patient. A rapid deterioration in health was observed in two patients with positive margins. These patients had a preoperative deranged renal function, precluding a complete removal of the tumor or kidney, resulting in positive margins. As a result, these patients showed a residual disease on follow-up imaging. Unfortunately, these patients died soon after the diagnosis of the residual disease because of chronic renal failure. One patient had a distant metastasis in the lung. He underwent video-assisted thoracoscopy and resection of the metastatic lesion. Presently, this patient is being treated with tyrosine kinase inhibitor therapy.

Four patients had postoperative complications. Among them, one patient had a wound infection, and another had wound dehiscence. Additionally, two patients (5.7%) had delayed urine leak. The preoperative RENAL score distribution, histologic characteristics, and Fuhrman grading are presented in Table 3. No tumors involved the renal sinus, and none of them had venous invasion at the time of surgery.

**Table 3:** Tumor characteristics

Tumor characteristics	N (%)
RENAL Score	
Low	26 (74.3%)
Medium	7 (20%)
High	2 (5.7%)
Pathological Stage	
T1	33 (94%)
T3	2 (5.7%)
Tumor Grade	
Grade 1	14 (40%)
Grade 2	16 (45%)
Grade 3	5 (14%)
Frozen Section Histology	
Negative	30 (85.7%)
Positive	2 (5.7%)
Not performed	3 (8.5%)
Margin status	
Positive	4 (11.4%)
Negative	31 (88.5%)
Histological Type	
Clear cell	25 (71%)
Papillary	6 (17%)
Multiloculated	2 (5.7%)
Angiomyolipoma	1 (2.8%)
Pseudotumors	1 (2.8%)

The median length of stay was four days (range: 3-7). The mean postoperative serum creatinine was 1.25 ± 1.4 mg/dL (range: 0.3-7.4 mg/dL) with only two cases (5.9%) of deranged renal function (creatinine > 1.5 mg/dL) as noted earlier. In our study, the mean serum creatinine after three months of surgery was 1.25 ± 1.4 mg/dL (range: 0.3-7.4 mg/dL), and no patient developed renal insufficiency post-surgery. Two cases (5.9%) of deranged renal function (i.e., creatinine > 2 mg/dL) had renal insufficiency prior to surgery with presurgery creatinine values of 4.56 and 6.23 mg/dL.

## Discussion

RN is the treatment of choice for renal tumors for more than 50 years [14] and is the standard against which all other surgical treatments for RCC are compared. RN is still performed in about 30% of cases of renal tumors.

With improvements in availability and cost of diagnostic imaging tools, more than 60% of renal tumors are now detected incidentally [15]. As a result, there is a reduction in stage and size of renal tumors at presentation. In a study conducted at the Memorial Sloan-Kettering Cancer Center, 80% of patients undergoing surgery for renal cancer had their tumors diagnosed incidentally [4]. In another study, 61% of the tumors were found incidentally during examinations performed for other reasons and unrelated to the patient’s presenting concerns [16]. In our study 23 cases (52.3%) were diagnosed incidentally, while 18 patients (40.9%) presented with abdominal or flank pain; one patient (2.3%) presented with lower urinary tract symptoms. This indicates that although a large group of patients presented with incidentally diagnosed renal masses during work-up for non-specific abdominal pain, there remain a large segment of late-presenting patients.

In nephrectomies for renal tumors, many surgeons prefer the extra-peritoneal flank incision above the 11th or 12th ribs [17]. We used the same incision in 57% of cases in our series. No comparative studies have been carried out focusing on the choice of incisions for PN or RN. In most circumstances, the choice of incision is up to the surgeon. In our study, in most of the procedures performed later in the series, we used anterior subcostal, transperitoneal incisions because, in our experience, there is no difference in the morbidity related to either of the incisions.

On the contrary, transperitoneal incisions provide quicker access to the renal pedicles, more room, and better exposure of the tumor. A mini-flank supra-12th rib incision was advocated by Wang [18] and Russo [19]. They concluded that PN by this incision provides the benefit of decreased morbidity while preserving renal parenchyma that would otherwise require more costly techniques of minimal access surgery. However, until further studies are available, both types of incisions remain well utilized.

Another controversial step during partial nephrectomy is the choice of cold or warm ischemia. In a study by Lane et al. [20], 660 solitary kidneys underwent partial nephrectomies, and ischemia was not an independent risk factor for the long-term deterioration of renal functions. They concluded the residual renal function depends on the quality and quantity of the residual renal parenchyma. We do not prioritize the type of ischemia, as we found no difference in the results. Partial nephrectomy is not associated with the development of chronic kidney disease [10], as supported by our findings.

The overall recurrence rates after NSS vary from 0 to 10.6% on long-term follow-up [21]. In a study by Shvero et al. [22], local recurrence was found in 2.8% of patients. In our study, a single distant metastasis was noticed in the lung at the 17-month follow-up. This patient had a 2.7-cm (pT1a) papillary RCC and clear margins on final histology. The time to metastasis, in this case, was relatively shorter for the tumor characteristics when compared with other studies. For instance, in a study by Levy et al. [23], a 7% recurrence rate was recorded and time to metastasis for T1 tumors was 38 months (range: 18-67), which is significantly longer than that observed in our study. A similar interval was observed in other studies [24]. However, the radiological investigations for follow-up remain inconsistent. This finding may support a more stringent imaging follow-up. Although there is no consensus on the follow-up protocols for renal tumors between various guidelines [25] for different tumor stages, our policy involves a baseline CT scan at the chest and abdomen levels, at the sixth and 12th month, then annually for up to five years. We also perform an abdominal ultrasound and a chest radiography at the 18-month follow-up.

The most common site of metastases is the lung [24], as also observed in our study. While we found no local recurrence, two patients had residual disease due to incomplete resection, as suggested by the positive tumor margins in the final histology. However, further resection in these patients was not possible due to physiological and anatomical reasons. FS histology was not performed in both these cases. A residual tumor in the PN bed was noted at six months in these patients, which emphasizes the importance of the availability of FS histology in centers practicing PN. However, whether FS is indeed essential while performing PN is not clear. In a study by Gordetsky et al. [26], 576 FS analyses were performed. On the final histopathology, 30 specimens (8.5%) had positive margins, of which four (13.3%) were classified as atypia, 17 (56.7%) as negative, and nine (30%) as positive on FS diagnosis. Intraoperative management was influenced in only seven cases. Therefore, the necessity and benefit of FS histology in PN have yet to be proven, but the aim of performing PN is to preserve residual renal function without compromising tumor control.

The survival rates with PN, especially for cT1 tumors, are similar to those achieved when RN is performed to treat similar tumors. Several studies have validated these findings. Badalato et al. [27] assessed 11,256 cases of pT1bN0M0 renal cortical tumors, which were identified in the SEER registry, having undergone PN or RN between 1998 and 2007, and no statistically significant difference in survival was identified after adjusting for different covariates like age, histological subtype, and grade of the tumors. Our survival rates with PN were similar to those reported in most studies. Patard et al. [28] reported in their study of 314 patients undergoing open partial nephrectomy the five-year disease-free survival rate was 97.8%. Lane and Gill reported similar results [29].

The complication rates associated with PN are generally higher than those of RN and range from 2.4% to 28% [30]. In our series, two patients developed postoperative grade IIIb complications (based on the Clavien-Dindo classification [CDC] in the form of persistent urinary leak that required double-J stent placement). One of these patients had a moderate RENAL score, and the other had a high RENAL score. Higher RENAL scores are associated with a higher number of postoperative complications. In a study by Reddy et al. [13], patients with moderate complexity renal lesions were found to develop more complications based on the CDC classification.

Our study was limited by its small sample size and nonstandard follow-up schedule. Therefore, factors determining the disease-free and overall survival could not be determined in our study. At this stage, however, we continue to collect data and evaluate our patient population via follow-up.

## Conclusions

PN is a viable option for patients with small renal tumors and has good survival rates in the intermediate follow-up. Further studies with larger sample sizes and longer follow-up schedules are needed to determine the factors governing the early postoperative complications and survival. Studies assessing the use of RENAL scoring to select patients with renal tumors who qualify for PN are also needed.
